# Collaborative digital platform France – Cuba: oncorehabilitation in reproductive and sexual health

**DOI:** 10.1186/s12909-021-02774-w

**Published:** 2021-06-09

**Authors:** Thierry Almont, Louis Bujan, Clarisse Joachim, Guillaume Joguet, Mylène Vestris, Rémi Houpert, Rodolfo Enriquez Rodriguez, Jaylin Carmenate, Beatriz Gutierrez, Yaima Galán, Line Kleinebreil, Christian Mésenge, Éric Huyghe, Jacqueline Véronique-Baudin

**Affiliations:** 1 Research on Cancer UF3596, Cancerology Department, Martinique University Hospital (CHU Martinique), Fort-de-France, Martinique; 2 General Cancer Registry of Martinique UF1441, Cancerology Department, Martinique University Hospital (CHU Martinique), Fort-de-France, Martinique; 3 French Education and Research Group in Andrology, Urology and Sexology (GEFRAUS), Reproductive Medicine Department, Paule de Viguier University Hospital, 330 avenue de Grande-Bretagne, TSA 70034, 31059, Toulouse cedex 9, France; 4 EA 3694 Human Fertility Research Group, Reproductive Medicine Department, Toulouse University Hospital, Paule de Viguier, 330 avenue de Grande-Bretagne, TSA 70034, 31059, Toulouse cedex 9, France; 5 Caribbean Center for Reproductive Medicine, CECOS CARAIBES, Pôle Parents-Enfants CHU de Pointe-à-Pitre 97159, Pointe-à-Pitre, Guadeloupe; 6 Hospital Ginecobstétrico Ramón González Coro, Calle 21 No. 854 e/. 4 y 6 Vedado, Plaza de la Revolución, 10400 La Habana, Cuba; 7 Registro Nacional de Cáncer de Cuba, Hospital Ginecobstétrico Ramón González Coro, Calle 21 No. 854 e/. 4 y 6 Vedado, Plaza de la Revolución, 10400 La Habana, Cuba; 8 World Francophone Digital University, UNFM / HNSM 14 rue du Val d’Osne, 94450 Saint-Maurice, France; 9 Francophone Association for Supportive Care (AFSOS), 76, rue Marcel Sembat, 33130 Bègles, France

**Keywords:** Oncosexuality, Oncofertility, Collaborative platform, Caribbean

## Abstract

**Background:**

In the French West-Indies, few studies have been performed on fertility and sexual problems in cancer survivors, which are frequent and recurring issues reported by surveys on unmet needs. Additionally, mutualizing human and material resources and promoting cooperation through a collaborative platform are the most appropriate response to complex health pathways in the Caribbean territories. Implementation of such a collaborative platform will help to launch a strategic Caribbean partnership to transfer theoretical and technical skills and care standards in oncofertility and oncosexuality.

**Methods:**

We propose to set up a collaborative digital platform to strengthen, from the French expertise, Cuban health professionals’ knowledge, know-how, and skills in oncofertility and oncosexuality. The project will be coordinated by a coordinating, scientific, and supervisory committee, and the main activities will include:

1. Theoretical training in e-learning adapted to low-speed Internet.
2. Practical training in fertility preservation and sexual rehabilitation.
3. Digital multidisciplinary consultation meetings for medical decisions to be taken for complex clinical cases.

The platform will benefit from a recurrent evaluation, by the two cancer registries of Martinique and Cuba, with the following performance indicators: number of Cuban professionals trained, number of professionals sensitized**,** hourly volumes (or number of training courses provided)***,*** satisfaction of trained professionals***,*** number of e-RCPs carried out online and number of missionaries supported. These indicators will be set up and analyzed by the registers.

This project meets the Cuban and French health policies (cancer plans and national sexual health strategies) and will be implemented in liaison with the Health Agencies of both countries and the Embassy of France in Cuba.

**Discussion:**

This project aims to provide support through bilateral exchanges to improve reproductive and sexual health in Cuba’s cancer patients. This collaboration will be based on a long-lasting French expertise and a solid Cuban health system. Consequently, this collaborative digital platform will contribute to data collection for cancer surveillance, and the two participating countries will ultimately be identified in the Caribbean as having centers of competence and excellence in oncofertility and oncosexuality with care standards.

## Background

Fertility and sexual problems are common and frequent issues reported by surveys on cancer survivors’ unmet needs [1–3].

Results of the French study VICAN, including 4174 cancer survivors, shows that, among 585 patients aged 18–40 at diagnosis, 57.8% of men and 67.4% of women did not discuss fertility preservation before treatment. Respectively, 79.3 and 77.2% did not perform cryopreservation [4].

The VICAN study shows that among the 2051 sexually active participants 2 years after diagnosis, 55.4% had substantial deterioration of their sexuality [5]. In a cross-sectional study including 1419 patients, 72.5% of men and 61.2% of women reported negative consequences of cancer and treatments on their sexual life [6].

No such studies have already been performed in the French West-Indies. However, sexual and reproductive issues could likely rank higher than in Metropolitan France, as some cancer sites have a higher incidence due to a genetic predisposition or environmental factors, which could make those cancers more aggressive [7–10].

The possibility to achieve a parental project and a satisfying intimate life is an essential element of quality of life, and oncofertility and oncosexuality care should be considered right from diagnosis throughout the cancer care pathway [1, 11].

Mutualizing human and material resources and promoting cooperation actions through a collaborative platform have been identified as the most appropriate and effective response to insularity challenges [12]. The university hospital of Martinique (CHUM) has decided to initiate a collaborative program for supporting the development of oncosexuality and oncofertility care in Ramón González Coro hospital in Cuba.

Implementation of this project will help to initiate a strategic Caribbean partnership for transferring theoretical and technical skills as well as standards of care in oncofertility and oncosexuality.

### Reproductive and sexual health policies

The French law applies in Martinique where fertility is legally regulated (article L.2141–11 of 6 August 2004, Public Health Code; Judgment of 3 August 2010, III-4.1) and institutionally organized (29 CECOS which are public centers for medically-assisted procreation (MAP)). Fertility preservation and specific care involving various disciplines such as oncologists, gynecologists, reproductive endocrinologists, and behavioral health practitioners [1, 13]. Fertility is targeted explicitly at objectives 7 and 8 of the last French cancer plan, notably Action 8.1, which aims to “*Ensure access to fertility preservation*” [14].

In Martinique, oncofertility care is implemented in collaboration with the CECOS CARAIBES, which is the West-Indies center for MAP located in Guadeloupe, and a local private center, which only provides fresh sperm cryopreservation up to date.

Oncosexuality has led to some public health actions with the first roadmap 2017–2020 of the French *National Sexual Health Strategy 2017–2030*. Specific interventions in oncosexuality are developed through Actions 18 and 21, providing to “*Take better account of relational and sexual life issues in consultations with patients suffering from chronic diseases and in therapeutic patient education programs*” and “*Adapt the planning of sexual and reproductive health provision for young people, considering the specific needs of the overseas territories and existing resources*”.

The CHUM has implemented oncosexuality care since April 2019 with Dr. Thierry ALMONT (T.A.), degreed in Epidemiology, Sexology, and MAP. He is currently the referent professional for seeing patients for their sexual problems.

To train other professionals to oncosexology, T.A. participated with Pr. Eric HUYGHE (E.H.: Urologist, Andrologist and Sexologist, Toulouse), Pr. Pascal BLANCHET, and Dr. Virginie ROUX (Urologists, Guadeloupe), to the implementation of a specific diploma in Sexology to be started late 2020 at the French West-Indies University.

In Cuba, the network of sexual and reproductive health services has national coverage. It is based on identifying infertile couples attending community primary healthcare centers for obstetricians, laboratory technicians, and psychologists to identify and treat the cause of their infertility. Community centers have the complete material and human resources to explore and treat infertility in couples, except for High Technology Assisted Reproduction (HTAR). HTAR is only available in the 4 high-technology reproduction centers of the country: one in Cienfuegos (Gustavo Aldereguía Lima Hospital), one in Holguín (Vladimir Ilich Lenin Hospital) and 2 in La Havana (Hermanos Ameijeiras and Ramón González Coro Hospitals). Cuban authorities plan to create two more centers composed of multidisciplinary teams of experts in reproduction (Gynecologists, Endocrinologists, Geneticists, Urologists, Biologists, Psychologists, and Laboratory Technicians), which will contribute to the development of laboratory techniques since 2017 [15], such as conventional in-vitro fertilization, intra-cytoplasmic in-vitro fertilization, gametes donation, testicular biopsy and cryopreservation of gametes and embryos.

The entire program is led by a National Commission for the infertile couple, under the Ministry of Public Health regulations.

### Cooperation and the hospital networks and partnerships program

To support cooperation initiatives, France has implemented a “*Hospital Networks and Partnerships*” (HNP) program for granting projects between a French hospital and a foreign hospital in a priority country identified in the program. This HNP is a joint-program signed by the French Agency for Development (AFD) and French Hospital Federation (FHF). In 2018, AFD allocated to FHF a 3-million-euro grant for implementing the 3rd HNP Project (called PRPH 3) for a period of 6 years.

Based on systems of peer-to-peer exchanges and mentoring, the collaborations established through the HNP enable partner hospitals to have a French “counterpart” able to understand and identify their needs and share its expertise.

The current project is granted through the French PRPH 3 programs to contribute to the improvement of the quality of oncofertility and oncosexuality care and management in Ramón González Coro Hospital in Cuba, with Pierre Zobda-Quitman Hospital in Martinique as the French counterpart partner.

## Methods

We propose to develop a collaborative digital platform adapted to Caribbean specificities (epidemiological, insular, cultural, etc.) to strengthen professional links and skills in oncofertility and oncosexology and reduce inequalities in access to reproductive and sexual health care for cancer patients.

The digital platform will be administered by a coordinating, a scientific and a supervisory committee and it will be implemented with the following key partners:

*Pierre Zobda-Quitman University Hospital* is the counterpart hospital. It is the most important administrative and care structure of the CHUM, hosting the Cancerology, Hematology and Urology Department and the Regional Cancer Registry of Martinique. It is the reference site for emergencies, medical specialties, complex surgical activities, and resuscitation.

*Ramón González Coro Hospital* is identified as the foreign partner hospital. Founded in 1971, it is a comprehensive national center for cancer, low-complexity MAP, pregnancy, prematurity, low-complexity surgery, genetics, heart diseases, and diabetes. The institutional activities mainly aim to coordinate continuing education, international cooperation, and access to comprehensive care.

*Hermanos Ameijeiras Hospital* is another comprehensive cancer center in Havana, guaranteeing highly specialized MAP through trans-disciplinary care and integration protocols in all medical and surgical specialties and the use of advanced technologies. It is identified as a “replication” center in which the Cuban Authorities will undertake the program’s achievements to increase territorial coverage of oncosexuality and oncofertility care.

Synergy partners are also identified as all other contributors to the project, which are:

*The CECOS CARAIBES* will be a comprehensive center for practical oncofertility training. It was created in 2007, then integrated into the French National Federation of CECOS in 2015. Its team is composed of 2 medical biologists and one laboratory technician. The center can provide, in carcinological, neurological, viral, or genetic contexts, preparation and preservation of embryos for adult couples and of gametes and germinal tissues for adolescents and adults.

*Paule de Viguier University Hospital* will be a center for practical training in oncofertility (CECOS Midi-Pyrénées) and oncosexuality (routine consultations). The CECOS Midi-Pyrénées provides cryopreservation of embryos, sperm, ovarian tissue, testicular tissue, and oocytes for adolescents, adults, prepubertal children, spinal cord injured, and viral risk patients.

Individual or couple sexology care are also provided by sexologist doctors and psychologists within the reproductive medicine department.

*The World Francophone Digital University (UNFM),* created in 2005, is based on the African Virtual University model. It aims (1) to maintain, through digital technology, a French presence in developing countries and in conflicts zones or areas of risk for cooperants, and (2) to build new relationships based on the sharing of values and expertise, particularly in former colonies.

The UNFM is made up of a multidisciplinary team capable of providing tailored e-learning courses to meet various demands for human or animal health, digital health, asset management, information technology, or journalism, for instance. The non-commercial system has been operational since 2005 and allows for creating online content worldwide that is fast, simple, inexpensive, and with broad access to low-speed Internet.

*The Regional Cancer Registry of Martinique (RCRM),* created in 1983, participates in the epidemiological surveillance of cancers by collecting and analyzing incidence and mortality data of patients usually living in Martinique at diagnosis. This contributes to understanding the evolution of cancers and implement efficient health policies. Data validated by the registry cover the period from 1981 to 31 December 2016.

The registry also contributes to research projects at the local, regional and national levels, allowing the valorization of collected and analyzed data towards public health actors. Besides, a scientific collaboration for cancer surveillance in the Caribbean via the Regional Network of Cancer Registries has been initiated at the West Indies-Guyana and Caribbean level, with the support of the International Agency for Research on Cancer (IARC).

*The National Cancer Registry of Cuba (NCR)* started in 1964 as part of the National Cancer Control Program of the National Institute of Oncology and Radiobiology (INOR) for cancer surveillance, prevention, and control strategy [16]. It collects, processes, and analyses data on all malignant neoplasms diagnosed in Cuba. Like the RCRM, the NCR is a population-based registry that contributes to international surveillance of cancer and provides reliable and high-quality data for the IARC’ Cancer Incidence in Five Continents’ and ‘International Childhood Cancer Incidence’ reports.

Both registries will recurrently assess the impact of the project’s activities with the following performance indicators: number of Cuban professionals trained, number of professionals sensitized, hourly volumes (or number of training courses provided), satisfaction of trained professionals, number of e-RCPs carried out online and number of missionaries supported. These indicators will be set up and analyzed by the two partner registers.

The collaborative platform will also be implemented in liaison with the complete support of the Health Agencies of both countries and the Embassy of France in Cuba, through the PRPH 3 program.

This project will follow three principal axes in order to create the collaborative platform:

1. To sensitize and inform healthcare professionals concerned (oncologists, organ specialists, reproductive biologists).

This ax aims to set-up professional education and development through e-learning and academic exchanges in reproductive and sexual health with the support of the World Francophone Digital University (UNFM). The pedagogical resources in sexology and MAP will be digitally-adapted for quality access even at very low Internet speeds, as is the case in Cuba and most Caribbean islands. The role and contribution of the UNFM to the project will consist of:

### Course registration

Teachers will record their courses with specific software from any Internet connection. Live compression will be processed during recording via the UNFM platform. The UNFM will provide remote assistance and support for software configuration, testing, and tutoring sessions.

### Web hosting

Hosting and creation of a dedicated responsive design website with the possibility to view a mini-biography of each speaker before the course. Multiple languages will be available with language selection for subtitling lectures and slide language.

Quizzes before and after each course will be an aid to learning, but also to update the program design.

A media library containing educational resources will be associated with the website for easy and permanent access.

Actions will be automatically traced according to specified indicators (number of participants, downloads, or views per resource, etc.)

### Examination and certification

A UNFM Certificate will be delivered after validation of the face-to-face examination in a Cuban University or a dedicated room at the MINSAP.

2. To help build the technical capacity of resource professionals for oncofertility and oncosexology care.

This specific objective consists of tailored practical training sessions and workshops during exchange missions in the CECOS (CARAIBES and Midi-Pyrénées) and Paule de Viguier university hospital. This training will lead to certification and will comprise:

### Interventions for male

Oncofertility sessions will focus on “preserving fertility from fresh sperm” and “preserving fertility from testicular sperm extraction” in the CECOS CARAIBES and on “fertility preservation from testicular tissue in adolescents and young adults” in the CECOS Midi-Pyrénées.

Oncosexuality sessions will focus on “sexual rehabilitation in prostate cancer patients” and “sexual rehabilitation in testicular cancer patients” at Paule de Viguier university hospital.

### Interventions for female

Oncofertility sessions will focus on “techniques for oocyte vitrification” in the CECOS Midi-Pyrénées and on “techniques for preserving fertility from ovarian tissue in adolescents and young adults” and “techniques for preserving fertility from in vitro matured oocytes” in the CECOS Midi-Pyrénées.

Oncosexuality sessions will focus on “sexual rehabilitation in breast cancer patients” and “sexual rehabilitation in cervix cancer patients” at Paule de Viguier university hospital.

3. To improve the quality of sexual and reproductive health care for complex cases. This specific objective consists of digital multidisciplinary consultation meetings (e-RCP) for medical decisions to be taken for complex clinical cases.

## Discussion

This project aims to provide support through bilateral exchanges, to improve reproductive and sexual health in cancer patients in Cuba. This collaboration will be based on a long-lasting French expertise and a solid Cuban health system.

In France, the first public MAP center was created in Paris in 1973 by Pr. Georges DAVID, a medical biologist and professor of histology and embryology. Since then, thanks to the efforts made by the learned cancer societies and the CECOS, fertility care has been structured with the first bioethics law in 1994, establishing the Biomedicine Agency (ABM), which controls MAP’s activities together with the regional health agencies. Then, with the successive cancer plans since 2003, access to fertility preservation before initiating cancer treatment is now guaranteed by Article L. 2141–11 of the law on bioethics.

The costs of preservation acts are listed in the nomenclature of medical biology procedures and are covered 100% by the Social Security. The storage period is not limited in time and the patient may request to use preserved gametes and tissues or to end the storage at any time.

Today, 29 public CECOS and 21 private centers are spread throughout the territory so that any patient can have easy access to a center. The 29 CECOS are located in university hospitals and are organized into a Federation. This Federation of the CECOS is a valuable asset for infertile couples or patients care, permanent and evolutionary reflection, and effective participation in fertility research. This organizational base has been reinforced with clinical practice guidelines for standardized care, with the first guidelines of the Francophone Association for Supportive Care (AFSOS) in 2010, updated in 2013 and 2019, and then the national practice guidelines currently being finalized by the INCa.

The French expertise of more than 45 years in fertility preservation is the foundation of assisting the development of fertility preservation in Cuba. Besides, 2 CECOS are partners in this project, including the CECOS Midi-Pyrénées coordinated by Pr. Louis BUJAN, a medical biologist who was president of the French Federation of CECOS from 2009 to 2015.

For sexuality, which has led to fewer organizational, legal and health measures than fertility, France has at least 20 years’ experience in this field with the first AFSOS practice guidelines for managing sexual disorders in cancer patients published in 2010 [17]. Since, medical initiatives have made it possible to build oncosexuality care on the existing CECOS network. This is for example the case of the CECOS in Toulouse, where the activity of reproductive medicine has diversified in parallel with fertility preservation to provide sexual health care (coordinated by Pr. Éric HUYGHE) for patients undergoing MAP or referred for fertility preservation. Besides these initiatives, various training courses in Sexology and Human Sexuality have been developed to give professionals the theoretical and clinical knowledge to evaluate, diagnose and treat sexual difficulties [18].

In support of this, the recent National Sexual Health Strategy 2017–2030 proposes a comprehensive approach to improve sexual and reproductive health, which aims to ensure that everyone has an independent, satisfying and safe sex life and that their sexual and reproductive rights are respected. In parallel, National practice guidelines for oncosexuality care are currently being finalized by the INCa.

With the French 45-year and 20-year official experience in oncofertility and oncosexuality, respectively, and the UNFM’s 15-year experience in providing e-learning resources accessible at very low Internet speeds, the collaborative platform we propose has a robust experiential foundation.

One of the means to ensure the operational quality is the follow-up and evaluation of the project and its activities by two qualified cancer registries. This project will allow for the first time to create a significant Martinique/Cuba cohort of cancer patients followed up for sexual and reproductive health. Subsequent relevant research protocols could be developed and related quality of life could be monitored based on standardized data from both countries’ cancer registries. Both registries have been cooperating since the 1980s and make recurring contributions to international epidemiology through reports produced by the IARC. The latest contribution was in Volume XI of the “*Cancer Incidence in Five Continents*” report [19]. As members of the Concord Group, they also contributed to the latest CONCORD III survivorship study [20].

With reliable health systems and expert partners, the collaborative platform aligns with both Martinique and Cuba’s sexual and reproductive health policies. Nevertheless, some factors could influence its implementation.

The first factor identified is the local Internet speeds and equipment available to Cuban professionals. While the common Internet access in Cuba is characterized by a low connection speed, limited bandwidth, and low rates of Internet penetration (5 to 25% in 2016 up to 40% in 2017, with only 5% of the population having full access to the Internet, usually through their places of work), the Cuban authorities provide easy access to high-speed Internet thanks to ADSL and the development of optical fiber, 3G and 4G, through Cuba’s telecommunications company ETECSA. Nevertheless, high-speed Internet access is mainly accessible to Cuban officials, health and academic institutions due to high cost and regulation policies [21–24].

To provide 7/7 access for Cuban professionals to digital training resources, several alternatives will be proposed, as:

1. the possibility of grouping together professionals on an institutional site where broadband is available;
2. the possibility to disable the video stream of training session for a better bandwidth;
3. the responsiveness of the platform so that its mobile version will also be accessible from a tablet or smartphone, since 62% of Cubans have cell phones (of which 93% are smartphones) [25];
4. the possibility to use a USB portable version of training resources of the platform in ultimate cases of broken Internet connection.

Another possible factor could be the time taken to obtain the visas for Cubans to come to France (for practical training sessions), as well as for French teams’ venue on Cuban territory (for the project implementation and follow-up). However, all the above-mentioned factors should have little or no impact, given the experience of the Cuban authorities in sharing big-data for cancer surveillance in the Caribbean with the IARC [15, 16], given the Cuban experience in international cooperation [12, 26, 27] and given the digital revolution in Cuba [21, 22, 25], notably with the implementation of Massive Online Open Courses by the University of Medical Sciences of Havana in cooperation with the Virtual Campus of Public Health of PAHO [22].

In addition to mutualization, training, and practice exchange, the main benefits of such a project are to improve quality of life through access to oncofertility and oncosexuality supportive care for all patients concerned; to systematize information and the prevention and management of sexual and reproductive sequelae of cancer treatments; to reduce the impact of cancer on personal life through fertility preservation and sexual rehabilitation; to ensure the quality, safety and pertinence of care at each stage of the patient’s personalized care program; and to globally improving sexual and reproductive health and help patients in the battle against cancer [28–33].

## Conclusion

Through the collaborative digital platform, French professionals will be able to share expertise in fertility preservation and sexual rehabilitation after cancer with Cuban healthcare professionals. This project complies with regulatory authorities of both countries. Related work will contribute to a continuum for data collection for cancer surveillance and the two participating countries will ultimately be identified in the Caribbean as having centers of competence and excellence in sexual and reproductive health with standardized practices guidelines and subsequent data. Such an initiative could be duplicated and spread throughout other Caribbean regions with qualified cancer registries.

## Data Availability

Not applicable.

## Abbreviations

ABM: Agence de Biomédecine (*Biomedicine Agency*)
AFD: Agence Française de Développement (*French Agency for Development*)
AFSOS: Association Francophone pour les Soins Oncologiques de Supprort (*Francophone Association for Supportive Care*)
CECOS: Centre d’Etude et de Conservation des Oeufs et du Sperme humains (*Center for the study and conservation of human eggs and sperm*)
CHUM: Centre Hospitalier Universitaire de la Martinique (*University Hospital of Martinique*)
ETECSA: Empresa de Telecomunicaciones de Cuba S.A. (*Cuba’s telecommunications company***)**
FHF: Fédération Hospitalière de France (*French Hospital Federation*)
HNP: Hospital Networks and Partnerships
HTAR: High Technology Assisted Reproduction
IARC: International Agency for Research on Cancer
INCa: Institut National du Cancer (*National Institute of Cancer*)
INOR: Instituto Nacional de Oncología y Radiobiología (*National Institute of Oncology and Radiobiology*)
MAP: Medically-Assisted Procreation
MINSAP: Ministerio de Salud Pública (*Ministry of Public Health*)
NCR: National Cancer Registry
PAHO: Pan American Health Organization
PRPH: Projet Réseaux et Partenariats Hospitaliers (*Hospital Networks and Partnerships Project*)
RCRM: Regional Cancer Registry of Martinique
UNFM: Université Numérique Fracophone Mondiale (*World Francophone Digital University*)
